# Prospective study of physical activity and risk of squamous cell carcinoma of the skin

**DOI:** 10.1186/1471-2407-11-516

**Published:** 2011-12-13

**Authors:** Petra H Lahmann, Anne Russell, Adèle C Green

**Affiliations:** 1 Queensland Institute of Medical Research, Cancer and Population Studies, 300 Herston Road, Herston, Brisbane 4006, Australia; 2 School of Translational Medicine, The University of Manchester, Manchester Academic Health Science Centre, Manchester, UK

## Abstract

**Background:**

The relationship between physical activity and risk of cutaneous squamous cell carcinoma (SCC) is unknown and difficult to investigate due to confounding by sun exposure. We prospectively examined the association of recreational and occupational physical activity and incidence of SCC accounting for photoaging and other risk factors.

**Methods:**

We used available information on physical activity from the Australian population-based Nambour Skin Cancer Study comprising 1,171 adults aged 25-75 years at baseline (1992). In sex-stratified analyses (person-based and tumor-based) we estimated the associations between type of activity and incidence of SCC prospectively to 2007.

**Results:**

During 16 years of follow-up, 98 men and 90 women newly developed SCC. We found no significant association between recreational activity measures and SCC after controlling for potential confounding factors including indicators of sun exposure. In men, the observed risk pattern was however suggestive of elevated risk with increasing total hours of recreational activity (compared to inactive men, RR (95%CI) 0.89 (0.54, 1.46) for ≤ 1.5 hrs/wk; 1.29 (0.82, 2.04) for ≤ 4.0 hrs/wk; 1.33 (0.86, 2.05) > 4.0 hrs/wk), while among women, higher level of occupational activity (standing and manual versus sedentary work activities) was associated with a reduced incidence of SCC tumors (*P *trend = 0.03).

**Conclusions:**

Despite some suggestion that recreational activity in men and occupational activity in women are related to occurrence of SCC, there is no firm support for a role of physical activity in the development of cutaneous SCC.

## Background

Squamous cell carcinoma (SCC) is the second most common skin cancer after basal cell carcinoma (BCC). High cumulative ultraviolet (UV) radiation exposure is the major environmental risk factor for SCC along with other host factors that are markers of UV susceptibility such as fair skin and fair/red hair [1-3].

Physical activity influences the risk of several types of cancer. Existing evidence strongly supports the role of regular physical activity in reducing risk of cancers of the colon, breast, and endometrium, and to a lesser extent cancers of the lung and pancreas [4]. It is also suggested that a similar association may exist for other cancers [5].

Proposed biological mechanisms through which physical activity may affect cancer risk or progression include changes in sex hormones, metabolic hormones and growth factors, inflammatory markers, and immune function [5,6]. Beneficial effects of physical activity are also conceivable for skin cancer, specifically those that improve immune function and those that increase detoxification of reactive oxygen species or increase DNA repair, thus reducing sun exposure-induced DNA damage [5,7,8].

Data on the association between physical activity and skin cancer are so scarce however [9], that conclusions on physical activity and body fatness as possible determinants of skin cancer could not be reached for lack of evidence [4]. A potential positive association between amount of recreational physical activity and skin cancer has been suggested in athletes, known to have very high UV radiation exposure levels while performing outdoor sports [10-12]. In addition to sun exposure, exercise-induced immunosuppression has also been suggested to increase risk of keratinocyte carcinomas and cutaneous melanoma in athletes [12,13].

The very few population-based studies on recreational or occupational physical activity and skin cancer incidence are confined to melanoma [9] or combined keratinocyte cancers (SCC and BCC) [14-16]. As for athletes, in the general population confounding of the association by sun exposure remains a potential explanation for any positive associations [16]. Evidence on occupational sun exposure as a factor associated with keratinocyte cancers remains unclear based on the few investigations exploring outdoor work or occupations in relation to these cancers [17-19].

Our aim was to examine the associations of recreational and occupational physical activity and incidence of SCC in a long-term community-based cohort study of skin cancer in Australia taking into account potential confounding by sun exposure and other established risk factors for SCC.

## Methods

### Study population and data collection

In this prospective study, 1,621 residents of the Queensland township of Nambour, who were originally randomly selected from the population-based electoral roll [20] and who participated in the Nambour Skin Cancer Prevention Trial (1992-1996) [21,22] were followed up until 2007. Ethical approval was obtained from the ethics committee of the Queensland Institute of Medical Research.

### Assessment and classification of physical activity measures

At baseline in 1992, participants underwent a physical examination (height and weight were measured by trained study personnel) and completed a health and fitness questionnaire including information on recreational physical activity, outdoor behavior, phenotype, previous skin cancer history (before 1992) and other personal characteristics including occupation.

#### Recreational physical activity

The physical activity questionnaire items were adapted from the National Health Survey 1989-90 [23,24]. Participants were asked the frequency and duration (in hours and minutes) of three categories of exercise done for sport, recreation or fitness during the past 2 weeks: 1) walking, 2) any exercise causing a moderate increase in heart rate or breathing, and 3) any exercise causing a large increase in heart rate or breathing. The latter two are referred to as moderate activity and vigorous activity in this study. Information was not recorded about the type of activities undertaken, but reported against each of the three categories above. Values for the individual categories were summed to derive total recreational activity reported here as hours per week (hrs/wk).

Metabolic equivalent (MET) values, defined as the ratio of work metabolic rate to a standard resting metabolic rate of 1.0 (4.184 kJ)/kg/hour were assigned for walking (light activity), moderate activity, and vigorous activity (3, 5, and 9 respectively) according to the *Compendium of Physical Activities *[25], and multiplied by the hours exercised at that intensity level per week, to obtain MET-hrs/wk of activity for each level and combined (total recreational activity).

All measures of recreational activity were used as categorical variables due to large proportions of zero values. The reference category for each variable included individuals who reported no activity, i.e. zero hours or minutes. Active study participants were ranked into approximately equal thirds based on sex-specific cut-points, except for the variable vigorous activity which had only two categories (non-active/active) due to small numbers of study participants practising vigorous sporting activities.

#### Occupational physical activity

Occupational physical activity was based on reported main lifetime occupations, coded according to the Australian Standard Classification of Occupations [26] and independently assigned by two of the authors (PHL, AR) as follows: sedentary, standing or manual (including heavy manual work).

### Endpoints and ascertainment of cases

Incident squamous cell carcinoma (SCC) was identified through detailed assessment procedures described previously [21,22,27]. All SCCs were verified histologically. Two outcomes were used in the analyses: (a) incidence of persons affected by new SCCs diagnosed after the baseline 1992 skin examination survey through to 31 December 2007, in the person-years of follow-up accumulated between these dates; (b) incidence of SCC tumors in the same person-years follow-up time as above. SCC tumors and person-years of follow-up were counted until date of withdrawal from the study, date of death or 31 December 2007, whichever came first. This analysis included 188 persons with new SCC, 98 men and 90 women.

### Statistical Analysis

For person-based analysis, relative risks (RRs) with 95% confidence intervals (95% CI) of SCC incidence for each categorical physical activity measure were estimated by generalised linear models, specifying Poisson distribution with a robust error variance [28] and person-years of follow-up as offset. For tumor-based analyses, RRs (95% CI) were derived using generalised linear models with negative binomial distribution. The negative binomial distribution has been recommended for analysing non-negative integer data which is overdispersed, usually due to aggregation of events within individuals [29]. All analyses were stratified by sex and multivariable models were simultaneously adjusted for the following established risk factors and potential confounders: age, history of skin cancer (before 1992), elastosis of the neck (clinical measure of photoaging), and freckling of the back. Treatment (betacarotene supplements and/or daily sunscreen) allocation in the trial 1992-1996 was included as a study design variable. We did not adjust for natural skin or hair colour, or tanning ability in the multivariable model because these innate characteristics were captured when degree of photoaging, a composite measure of sun sensitivity and cumulative sun exposure, was included in the model [30].

To test for linear trend across levels of physical activity, we modelled the sex-specific median value in each category as a continuous variable. To examine the potential effect modification of the physical activity-SCC association by sex or history of skin cancer, interaction terms for total recreational activity with sex or skin cancer history were tested. A *P *value for interaction was calculated, referring to the interaction term of the sex variable or the skin cancer history variable and total recreational activity (categorical) over the entire cohort. Neither of the interaction terms was statistically significant (*P *> 0.05). A sex-stratified analysis was performed due to expected differences in physical activity patterns between men and women.

## Results

For the present study, participants with missing data on physical activity items (n = 282) and body mass index (BMI, kg/m^2^) (n = 168) were excluded, leaving an analytical cohort of 1,171 men and women. Participants who were excluded from the analysis tended to be younger than their included counterparts, but the difference in mean age at baseline was only statistically significant in men (47.2 vs 50.5 yrs). Further, excluded women were more likely to have a lower education, while excluded men were more likely to have a lower grading of clinical elastosis and were less likely to have a keratinocyte cancer prior to 1992 compared to their respective counterparts included in the study.

Study participants aged 25 to 75 years at baseline (1992) were followed for an average period of 14.4 (± 3.8) years, yielding a total of 16 887 person-years. Characteristics of the study population stratified by case status and sex are presented in Table 1, 2. Compared to those unaffected, persons with SCC were on average older, more likely to burn than tan on strong sun exposure, have a higher grade of freckling on the back, and severe clinical elastosis of the neck (Table 1). Study participants with or without SCC did not differ in their leisure or occupational sun exposure in 1992 or their cumulative sun exposure over time (data not shown). Twenty-eight percent had a history of keratinocyte cancer (SCC and/or BCC) before the baseline examination in 1992.

**Table 1:** Characteristics by skin cancer status, the Nambour Skin Cancer Study (N = 1,171)

Characteristics		Women			Men	
	
	SCC (n = 90)	No SCC (n = 575)	p-value	SCC (n = 98)	No SCC (n = 408)	p-value
	*Mean (SD)*					
Age (years)	57.3 (9.9)	48.1 (12.1)	< 0.001	59.7 (11.7)	48.3 (13.0)	< 0.001
BMI, kg/m^2^	26.2 (5.0)	25.8 (4.7)	0.515	26.6 (3.8)	26.5 (3.6)	0.868
	*N (%)*					
History of skin cancer	48 (53.3)	123 (21.4)	< 0.001	64 (65.3)	88 (21.6)	< 0.001
Education						
High school only	63 (70.0)	366 (63.7)	0.446	37 (37.8)	167 (40.9)	0.284
Certificate/diploma	3 (3.3)	34 (5.9)		31 (31.6)	147 (36.0)	
University	22 (24.4)	147 (25.6)		22 (22.4)	59 (14.5)	
Trade/other	2 (2.2)	28 (4.9)		8 (8.2)	35 (8.6)	
Pack-years of smoking						
None	56 (62.2)	383 (66.6)	0.599	35 (35.7)	181 (44.4)	0.115
1-7	13 (14.4)	92 (16.0)		16 (16.3)	65 (15.9)	
> 7-20	10 (11.1)	49 (8.5)		13 (13.3)	67 (16.4)	
> 20	11 (12.2)	51 (8.9)		34 (34.7)	95 (23.3)	
Tanning ability of skin						
Always burn	33 (36.7)	130 (22.6)	0.003	29 (29.6)	55 (13.5)	< 0.001
Burn then tan	54 (60.0)	377 (65.6)		62 (63.3)	310 (76.2)	
Only tan	3 (3.3)	68 (11.8)		7 (7.1)	42 (10.3)	
Painful sunburns throughout life (1992)						
Never	10 (11.1)	62 (10.8)	0.084	15 (15.3)	48 (11.8)	0.798
Once	21 (23.3)	114 (19.8)		14 (14.3)	55 (13.5)	
2-5 times	29 (32.2)	263 (45.7)		40 (40.8)	176 (43.2)	
More than 5 times	30 (33.3)	136 (23.7)		29 (29.6)	128 (31.4)	
Freckling of the back (1992)^a^						
Nil	18 (20.5)	197 (35.1)	0.001	19 (19.6)	125 (32.0)	< 0.001
Mild	29 (33.0)	215 (38.3)		25 (25.8)	137 (35.0)	
Moderate	30 (34.1)	103 (18.3)		20 (20.6)	76 (19.4)	
Severe	11 (12.5)	47 (8.4)		33 (34.0)	53 (13.6)	
Clinical elastosis of the neck (1992)^a^						
Nil	5 (5.6)	160 (27.8)	< 0.001	2 (2.1)	72 (17.7)	< 0.001
Mild	38 (42.2)	282 (49.0)		35 (36.1)	205 (50.4)	
Severe	47 (52.2)	133 (23.1)		60 (61.9)	130 (31.9)	

^a ^Column percentages may not sum to 100% due to missing values (freckling of the back: women n = 15, men n = 18; elastosis: men n = 2)

**Table 2:** Type of physical activity by skin cancer status, the Nambour Skin Cancer Study (N = 1,171)

			Women			Men	
**Type of physical activity**		**SCC (n = 90)**	**No SCC (n = 675)**	**p-value**	**SCC (n = 98)**	**No SCC (n = 408)**	**p-value**

Recreational activity, total (hrs/wk)							
None		32 (35.6)	190 (33.0)	0.823	33 (33.7)	169(41.4)	0.093
F ≤ 1	M ≤ 1.5	18 (20.0)	135 (23.5)		17 (17.3)	88 (21.6)	
F ≤ 3	M ≤ 4	19 (21.1)	131 (22.8)		21 (21.4)	81 (19.9)	
F > 3	M > 4	21 (23.3)	119 (20.7)		27 (27.6)	70 (17.2)	
Walking (hrs/wk)							
None		40 (44.4)	278 (48.3)	0.479	43 (43.9)	261 (64.0)	< 0.001
F < 1	M < 1.2	17 (18.9)	88 (15.3)		11 (11.2)	57 (14.0)	
F < 2.5	M < 2.5	13 (14.4)	107 (18.6)		20 (20.4)	46 (11.3)	
F ≥ 2.5	M ≥ 2.5	20 (22.2)	102 (17.7)		24 (24.5)	44 (10.8)	
Moderate activity (hrs/wk)							
None		74 (82.2)	434 (75.5)	0.374	72 (73.5)	290 (71.1)	0.895
F < 1.5	M < 1.75	8 (8.9)	72 (12.5)		13 (13.3)	59 (14.5)	
F ≥ 1.5	M ≥ 1.75	8 (8.9)	69 (12.0)		13 (13.3)	59 (14.5)	
Vigorous activity (hrs/wk)							
None		85 (94.4)	527 (91.7)	0.363	92 (93.9)	350 (85.8)	0.030
F ≤ 8	M ≤ 12	5 (5.6)	48 (8.3)		6 (6.1)	58 (14.2)	
Recreational activity, total (MET-hr/wk)							
None		32 (35.6)	190 (33.0)	0.911	33 (33.7)	169 (41.4)	0.417
F ≤ 3.7	M ≤ 6	19 (21.1)	132 (23.0)		19 (19.4)	81 (19.9)	
F ≤ 10.7	M ≤ 18	21 (23.3)	125 (21.7)		23 (23.5)	82 (20.1)	
F > 10.7	M > 18	18 (20.0)	128 (22.3)		23 (23.5)	76 (18.6)	
Occupational activity							
Sedentary		25 (29.8)	192 (37.4)	0.073	23 (24.2)	104 (29.1)	0.200
Standing		9 (10.7)	84 (16.3)		37 (38.9)	146 (40.8)	
Manual		50 (59.5)	238 (46.3)		35 (36.8)	108 (30.2)	

At baseline, 33% of all women and 40% of all men were classified as physically inactive, i.e. they did not report any time spent walking or performing more moderate/vigorous recreational activities. Among physically active study participants, men reported more hours of total recreational activity than women. On average, active men (n = 304) spent a mean (SD) of 4.2 (4.5) hrs/wk and active women (n = 443) 2.9 (3.3) hrs/wk on any recreational activity. Walking was the predominant activity (men: 2.3 (3.7); women 1.9 (2.8) hrs/wk).

Women did not differ by case status in any of the recreational physical activity measures, whereas males with SCC reported more hours of walking, but less hours of vigorous exercise, compared to their counterparts with no SCC (Table 2). Regarding occupational activity, affected females tended to have a higher proportion of manual activity and lower proportion of sedentary occupations than nonaffected, but this difference did not reach statistical significance. Overall, women in manual activity tended to do their work mainly indoors (45%) or both indoors and outdoors (47%), whereas men in manual activity performed their work predominantly outdoors (66%).

In age-adjusted and multivariable-adjusted models, neither total recreational physical activity nor any individual type of activity or MET-hrs were significantly associated with risk of SCC when analysed by persons (Table 3) or tumor counts (Tables 4) in both sexes.

**Table 3:** Relative risks (RRs) and 95% CI of SCC (1992-2007) according to type of physical activity, stratified by sex

Type of physical activity		Women with SCC/Total	Age-adjusted RR (95% CI)	Multivariable^a ^RR (95% CI)	Men with SCC/Total	Age-adjusted RR (95% CI)	Multivariable^a ^RR (95% CI)
Recreational activity, total (hrs/wk)			(n = 665)	(n = 650)		(n = 506)	(n = 486)
None		32/222	Reference	Reference	33/202	Reference	Reference
F ≤ 1	M ≤ 1.5	18/153	0.88 (0.53, 1.48)	0.92 (0.56, 1.53)	17/105	0.82 (0.50, 1.36)	0.89 (0.54, 1.46)
F ≤ 3	M ≤ 4	19/150	0.96 (0.57, 1.60)	1.04 (0.62, 1.74)	21/102	1.23 (0.78, 1.94)	1.29 (0.82, 2.04)
F > 3	M > 4	21/140	0.87 (0.53, 1.41)	0.85 (0.52, 1.38)	27/97	1.45 (0.93, 2.25)	1.33 (0.86, 2.05)
p-trend			0.647	0.557		0.053	0.135
Walking (hrs/wk)							
None		40/318	Reference	Reference	43/304	Reference	Reference
F < 1	M < 1.2	17/105	1.30 (0.81, 2.10)	1.36 (0.85, 2.17)	11/68	0.99 (0.56, 1.74)	1.04 (0.59, 1.82)
F < 2.5	M < 3.5	13/120	0.81 (0.44, 1.46)	0.99 (0.55, 1.78)	20/66	1.61 (1.06, 2.47)	1.64 (1.07, 2.51)
F ≥ 2.5	M ≥ 3.5	20/122	0.98 (0.60, 1.62)	1.06 (0.65, 1.74)	24/68	1.63 (1.06, 2.50)	1.37 (0.90, 2.08)
p-trend			0.734	0.997		0.026	0.142
Moderate activity (hrs/wk)							
None		74/508	Reference	Reference	72/362	Reference	Reference
F < 1.5	M < 1.7	8/80	0.72 (0.37, 1.43)	0.67 (0.35, 1.29)	13/72	0.88 (0.52, 1.48)	0.95 (0.58, 1.57)
F ≥ 1.5	M ≥ 1.7	8/77	0.79 (0.41, 1.51)	0.66 (0.35, 1.27)	13/72	0.96 (0.58, 1.60)	1.05 (0.64, 1.70)
p-trend			0.389	0.141		0.859	0.868
Vigorous activity (hrs/wk)							
None		85/612	Reference	Reference	92/442	Reference	Reference
F ≤ 8	M ≤ 12	5/53	1.49 (0.69, 3.21)	1.30 (0.63, 2.65)	6/64	1.02 (0.47, 2.21)	1.08 (0.54, 2.18)
p-value			0.410	0.533		0.953	0.831
Recreational activity, total (MET-hr/wk)							
Sedentary		32/222	Reference	Reference	33/202	Reference	Reference
F ≤ 3.7	M ≤ 6	19/151	0.98 (0.55, 1.52)	0.96 (0.58, 1.57)	19/100	0.93 (0.58, 1.50)	1.01 (0.63, 1.61)
F ≤10.7	M ≤ 18	21/146	0.96 (0.58, 1.58)	1.07 (0.64, 1.81)	23/105	1.16 (0.74, 1.82)	1.16 (0.74, 1.83)
F > 10.7	M > 18	18/146	0.82 (0.50, 1.37)	0.78 (0.47, 1.30)	23/99	1.41 (0.88, 2.23)	1.31 (0.84, 2.06)
p-trend			0.481	0.318		0.114	0.208
Occupational activity							
Sedentary		25/217	Reference	Reference	23/127	Reference	Reference
Standing		9/93	0.85 (0.55, 1.32)	0.69 (0.46, 1.05)	37/183	0.84 (0.54, 1.30)	0.89 (0.58, 1.36)
Manual		50/288	0.70 (0.37, 1.34)	0.64 (0.33, 1.24)	35/143	1.00 (0.68, 1.48)	1.13 (0.76, 1.69)
p-value			0.456	0.131		0.663	0.532

^a ^Model adjusted for age (years), treatment allocation, elastosis of the neck, freckling of the back and skin cancer history

**Table 4:** Relative risks (RRs) and 95% CI of SCC counts (1992-2007) according to type of physical activity, stratified by sex

		Women			Men		
		
Type of physical activity		Tumour count	Age-adjusted RR (95% CI)	Multivariable^a ^RR (95% CI)	Tumour count	Age-adjusted RR (95% CI)	Multivariable^a ^RR (95% CI)
Recreational activity, total (hrs/wk)			(n = 665)	(n = 650)		(n = 506)	(n = 486)
None		62	Reference	Reference	59	Reference	Reference
F ≤ 1	M ≤ 1.5	27	0.79 (0.42, 1.49)	0.89 (0.48, 1.65)	57	1.09 (0.58, 2.07)	1.03 (0.55, 1.94)
F ≤ 3	M ≤ 4	29	0.91 (0.48, 1.70)	0.97 (0.52, 1.79)	41	1.18 (0.60, 2.32)	1.20 (0.61, 2.35)
F > 3	M > 4	31	0.75 (0.41, 1.39)	0.76 (0.42, 1.38)	62	1.77 (0.93, 3.37)	1.71 (0.91, 3.21)
p-trend			0.454	0.409		0.073	0.076
Walking (hrs/wk)							
None		74	Reference	Reference	78	Reference	Reference
F < 1	M < 1.2	28	1.21 (0.64, 2.26)	1.41 (0.76, 2.62)	30	1.06 (0.52, 2.20)	1.40 (0.68, 2.89)
F < 2.5	M < 3.5	14	0.60 (0.29, 1.25)	0.72 (0.35, 1.48)	52	1.99 (1.02, 3.90)	1.93 (0.99, 3.76)
F ≥ 2.5	M ≥ 3.5	33	1.06 (0.59, 1.90)	1.12 (0.63, 2.01)	59	1.99 (1.04, 3.81)	1.59 (0.85, 2.98)
p-trend			0.945	0.921		0.027	0.149
Moderate activity^1 ^(hrs/wk)							
None		121	Reference	Reference	157	Reference	Reference
F < 1.5	M < 1.7	17	1.00 (0.50, 2.02)	0.83 (0.42, 1.65)	33	1.18 (0.59, 2.38)	1.00 (0.52, 1.92)
F ≥ 1.5	M ≥ 1.7	11	0.66 (0.29, 1.50)	0.60 (0.27, 1.34)	29	0.95 (0.46, 1.96)	1.22 (0.59, 2.51)
p-trend			0.325	0.192		0.912	0.599
Vigorous activity^2 ^(hrs/wk)							
None		144	Reference	Reference	211	Reference	Reference
F ≤ 8	M ≤ 12	5	1.03 (0.32, 3.27)	0.90 (0.29, 2.85)	8	0.78 (0.29, 2.09)	0.73 (0.27, 1.96)
p-value			0.961	0.861		0.626	0.534
Recreational activity, total (MET-hr/wk)							
Sedentary		62	Reference	Reference		Reference	Reference
F ≤ 3.7	M ≤ 6	28	0.81 (0.43, 1.51)	0.91 (0.49 1.68)	59	1.05 (0.55, 2.02)	1.02 (0.54, 1.94)
F ≤ 10.7	M ≤ 18	31	0.89 (0.48, 1.65)	0.98 (0.53, 1.82)	54	1.37 (0.72, 2.60)	1.35 (0.72, 2.53)
F > 10.7	M > 18	28	0.74 (0.40, 1.39)	0.73 (0.40, 1.33)	57	1.64 (0.83, 3.23)	1.60 (0.83, 3.09)
p-trend			0.438	0.320	49	0.125	0.134
Occupational activity							
Sedentary		35	Reference	Reference		Reference	Reference
Standing		11	0.72 (0.42, 1.23)	0.53 (0.32, 0.91)	52	0.89 (0.49, 1.61)	0.92 (0.51, 1.65)
Manual		96	0.46 (0.22, 1.10)	0.48 (0.22, 1.07)	72	0.89 (0.51, 1.53)	0.90 (0.53, 1.53)
p-value			0.155	0.027	84	0.891	0.925

^a ^Model adjusted for age (years), treatment allocation, elastosis of the neck, freckling of the back and skin cancer history

Among men, the risk estimates for total recreational activity and walking suggested a slightly increased risk in SCC, but they were not statistically significant (except for walking 1.2-3.5 hrs/wk, person-based) and there were no dose-response trends. Occupational activity was not significantly related to SCC in men, but was in women. With increasing work activity, women had a reduced incidence of SCC (*P *trend = 0.03) in the tumor-based analysis. This association did not reach statistical significance in the person-based analysis.

In additional analyses (data not shown), neither stratification of tumors according to whether they occurred on chronically or occasionally sun-exposed skin sites, nor mutual adjustment of recreational and occupational activity materially affected the risk estimates in the respective physical activity models, with one exception. In men, the recreational activity-SCC association based on tumor counts was slightly stronger after adjustment for occupational activity (RR (95%CI) low, medium, high vs none: 1.36 (0.74, 2.49), 1.55 (0.81-2.96), 2.01 (1.09-3.69); p*_trend _*= 0.032). Finally, for completeness, we assessed the use of sunscreen (non-user/irregular user/regular user) and found no significant difference in sunscreen use across levels of recreational or occupational activity in either men or women, though compared with inactive women, women with active recreations tended to be more regular sunscreen users (data not shown).

## Discussion

In this large prospective study of Australian adults, recreational physical activity was not significantly associated with development of cutaneous SCC when adjusted for other risk factors including indicators of sun exposure. In men, we observed a suggestive pattern of elevated risk with increasing hours of total activity or walking, but trends did not reach statistical significance. In contrast to recreational activity, higher occupational activity, i.e. standing or manual activity, was significantly associated with reduced risk of SCC among women in tumor-based, but not in person-based analysis.

To our knowledge, this study is the first to investigate the association between physical activity, both recreational and occupational, and cutaneous SCC and therefore, comparisons with other relevant reports are limited. Our observation of a suggestive positive association for recreational activity in men corroborates the finding from a large Danish prospective study [16] showing a highly significant positive association of leisure activity with keratinocyte cancers in most active men, but not in women. Schnohr and colleagues [16] speculated that the sex difference found in their study may be due to more outdoor exercise, less clothing, and less use of sunscreen in men than women. In their study on risk of cancer in general, the leisure activity-skin cancer association was not adjusted for outdoor sun exposure or any proxy measure reflecting sun exposure, and thus may be confounded by UV radiation. In contrast, in our SCC-study, we accounted for clinical elastosis, a marker of photoaging due to cumulative sun exposure [30]. This may explain the lack of an independent effect of any of the recreational activity measures on SCC in our data.

Findings from two other reports examining incidence of melanoma or keratinocyte cancers in women with higher versus lower recreational activity during adult life are equivocal. In a study of female college alumnae from the US, there was no difference in the lifetime occurrence of keratinocyte cancers and melanomas between former college athletes and nonathletes [31]. A Finnish register-based study of life-long physical activity and cancer risk among female teachers indicated that teachers of physical education had a non-significant 2-fold higher standardized incidence ratio of melanoma compared to language teachers during the follow-up period 1967-91 [15]. Both investigations lack adjustment for sun exposure or any other potential confounder.

In the present study, physical activity at work was inversely and significantly associated with the development of SCC in women when tumor counts where considered. The person-based analysis suggested a similar protective effect with higher work activity. The lack of an association among men may be related to the overall higher proportion of manual activity in women (48% vs 32%) which was predominantly performed indoors and included home duties, in contrast to men. Thus, some potentially beneficial effects of a higher activity level combined with lower outdoor sun exposure may have contributed to the reduced risk in women. Results from a large hospital-based case-control study of selected cancer sites indicated no effect of work activity assessed as energy expenditure and sitting time during work hours on risk of keratinocyte cancers [14]. Compatible with our investigation, the assessment of occupational activity level was based on job titles, but the cancer outcome was SCC and BCC combined, and sun exposure was not accounted for. Except for this single study [14], we found no other relevant evidence in the literature on occupational activity and risk of SCC.

Similar to risk of internal cancers, any reduction in risk of SCC in persons with higher physical activity levels may be due to compensatory mechanisms by the physical activity, i.e. beneficial effects, specifically when sun-exposure is accounted for as in our study. However, this cannot fully explain the opposite tendency, i.e. towards a positive association between recreational activity and SCC in men observed in this or one other study [16]. Further, it is not fully understood yet how much and what type of physical activity is required to diminish risk of different cancer types [4,32]. Many of the metabolic changes induced by physical activity are mediated through reduced adiposity and effects on other body compartments (e.g. skeletal muscle), and therefore it remains to be determined whether physical activity acts independently of weight control or not [5,7].

Some limitations warrant consideration when interpreting results of our study. Due to missing information on physical activity and BMI, we excluded 450 (28%) participants in the original study cohort which potentially could lead to selection bias. When we compared other study characteristics between those with and without missing data however, we found that the main differences were among men only: men who were excluded were on average 3 years younger than those who were included, and this was reflected in the lower actinic damage in the former. Given the lack of differences between women and the lack of any major differences among men who were excluded or included, we believe that any selection bias would be minimal.

The assessment of recreational physical activity relied on self-reports and referred to a time period of two weeks prior to the baseline examination which may not reflect physical activity patterns over an extended time period. However, the questionnaire activity items and the reference time period were adapted from the 1989-90 National Health Survey (NHS) [24] and considered reliable measures. In this study 64% of the participants reported some form of recreational activity which is similar to the proportion (65%) of Queensland adults who reported physical exercise in the 1989-90 NHS [24]. Among Nambour study participants the proportion of people walking was somewhat higher (51% vs 42%), and lower for moderate (24% vs 36%) or vigorous (12% vs 17%) activity compared to Queensland data from the NHS. While recreational activity patterns and thus intensity may have differed, the overall level of activity or inactivity in this study was compatible with national data.

Regular sunscreen use has been shown to reduce the occurrence of SCC in this study population [22,33], and thus we routinely adjusted for treatment allocation in all analyses. The known influence of fair skin on both sunscreen use and skin cancer [34] was also accounted for in all analyses and should not have affected results. Further, baseline (1992) sunscreen use was not significantly correlated with recreational or occupational physical activity in men or women and did not differ by case status. The tendency of women to be more regular users with higher recreational activity levels compared to inactive ones observed here and elsewhere [35] would be expected to reinforce an inverse association between physical activity and SCC if it were present, thus lending further weight to the null findings.

## Conclusions

In summary, this prospective study in Australian middle-aged adults provides little evidence for the role of recreational or occupational activity in SCC occurrence. Our data are suggestive of a positive association between total hours of recreational activity and SCC in men and show an isolated protective effect of high work activity in women, but these associations are not robust. Since this is the first study on physical activity and SCC, more comparative data are needed to support or refute our findings.

## Competing interests

The authors declare that they have no competing interests.

## Authors' contributions

PHL conceived the analysis plan and design, provided technical assistance to statistical analysis, interpreted the data and drafted the manuscript. AR carried out the statistical analysis and participated in the design of the study. ACG conceived of the study, participated in its coordination and helped to draft the manuscript. All authors read and approved the final manuscript.

## Pre-publication history

The pre-publication history for this paper can be accessed here:

http://www.biomedcentral.com/1471-2407/11/516/prepub
